# Return to Play After Distal Biceps Tendon Repair

**DOI:** 10.1007/s12178-022-09742-x

**Published:** 2022-02-23

**Authors:** Luis F Carrazana-Suarez, Sean Cooke, Christopher C. Schmidt

**Affiliations:** 1 Department of Orthopaedic Surgery, University of Pittsburgh Medical Center, 9104 Babcock Blvd, Suite 5113, Pittsburgh, PA 15237 USA; 2 Shoulder and Elbow Mechanical Research Laboratory, Department of Orthopaedic Surgery, University of Pittsburgh Medical Center, Pittsburgh, PA USA; 3 Department of Mechanical Engineering and Material Science, University of Pittsburgh, Pittsburgh, PA USA

**Keywords:** Distal biceps, Biceps tear, Distal biceps repair, Return to play, Sports injuries, Outcomes

## Abstract

**Purpose of Review:**

Distal biceps tendon ruptures (DBTR) are uncommon injuries in 40- to 50-year-old men but occur at a younger age in the athlete population. The distal biceps tendon is an important supinator of the forearm and flexor of the elbow. A complete injury results in limiting function in the upper extremity. The current review evaluates the different options in management and the current literature on return to play in athletes.

**Recent Findings:**

The distal biceps tendon inserts on the posterior aspect of the radial tuberosity as two independent heads. The long head footprint is more proximal and posterior giving it a better lever arm for supination. The short head footprint is more distal and anterior giving it a better lever arm for flexion. Surgical anatomic repair is highly recommended among the athlete population, to restore proper function of the upper extremity. There is scarce literature on return to play among athletes. The most recent studies on high-performance athletes are on National Football League (NFL) players. These studies showed that 84–94% of NFL players returned to play at least one game after distal biceps repair. Compared to matched control groups, there was no difference in the player’s performance after surgery.

**Summary:**

Anatomic repair of DBTR results in excellent outcomes, high return to work, and high rate of return to play among athletes. When compared to matched control groups, NFL players have the performance score and play the same number of games after surgery.

**Supplementary Information:**

The online version contains supplementary material available at 10.1007/s12178-022-09742-x.

## Introduction

The biceps brachii muscle is the primary supinator of the forearm and secondary flexor of the elbow [1, 2]. Rupture to the distal biceps tendinous insertion make up 3–10% of all biceps injuries and has an incidence of up to 5.35 cases per 100,000 per year [3–5]. These injuries are common among 40- to 50-year-old men and 86% of the time affects the dominant extremity [6, 7]. In athletes and active patients, surgical repair is recommended, due to devastating limitations in upper extremity function. Patients can experience cramping with activity and loss of supination and flexion strength. After nonoperative treatment of a complete distal biceps tendon rupture (DBTR), Morrey et al. [8] reported 40% loss of supination strength and 30% loss of flexion strength. Another study showed that maximum supination strength decreases on average 40% (range, 26–60%) and maximum flexion strength decreases on average 20% (range, 0–40%) [9].

As the primary supinator of the forearm, after a ruptured biceps, supination torque, power, and endurance are dependent solely on the brachioradialis and supinator muscles [10]. The brachioradialis muscle supinates the forearm from pronated to neutral position but becomes a forearm pronator from neutral to terminal supination. Also, the supinator muscle moment loses its moment arm as the forearm supinates. Neither muscle can compensate for a complete DBTR in neutral to terminal supination [10]. This deficit becomes more obvious when trying to supinate away from the body, without the support of the trunk (e.g., hitting a baseball, swinging a golf club, holding a rifle).

Distal biceps tendon ruptures (DBTR) typically occur after an eccentric load is applied while the elbow is flexed and the forearm is supinated [6, 11]. Regardless of fixation technique, surgical reattachment through either anterior or posterior approach results in high patient satisfaction, low pain levels, and good to excellent outcomes [4, 9, 12–20]. In the last 10–15 years, there has been an improved understanding of the anatomy and the biomechanics of the distal biceps leading to improved performance [2, 21–29]. However, return to play and performance in athletes have been under investigated. The purpose of the current review is to present the literature on DBTR and return to play and outcomes in athletes after this injury.

## Anatomy

**Figure 1:**
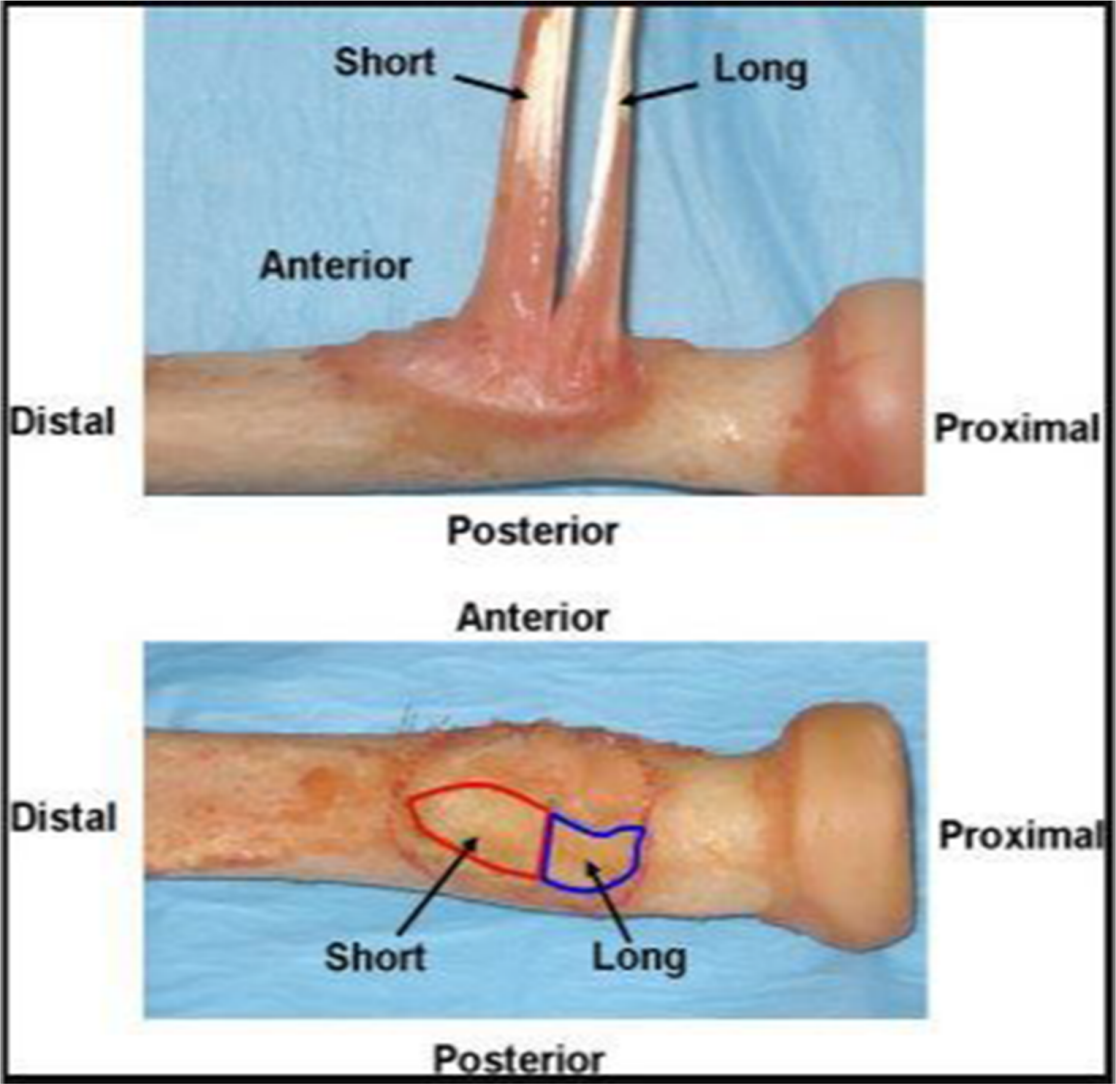
The top picture shows the two distinct heads of the distal biceps tendon, the short and long heads. The bottom picture illustrates the footprint of each head. Notice that the short head is more distal and occupies the apex of the bicipital tuberosity while the long head is more proximal and posterior. (Reprinted from *J Shoulder and Elbow Surgery*, 2012, doi.org/10.1016/j.jse.2011.04.030, Jarrett CD, Weir DM, Stuffmann ES, Miller MC, Schmidt CC with permission from Elsevier) (Anatomic and biomechanical analysis of the short and long head components of the distal biceps tendon—ScienceDirect)

**Figure 2:**
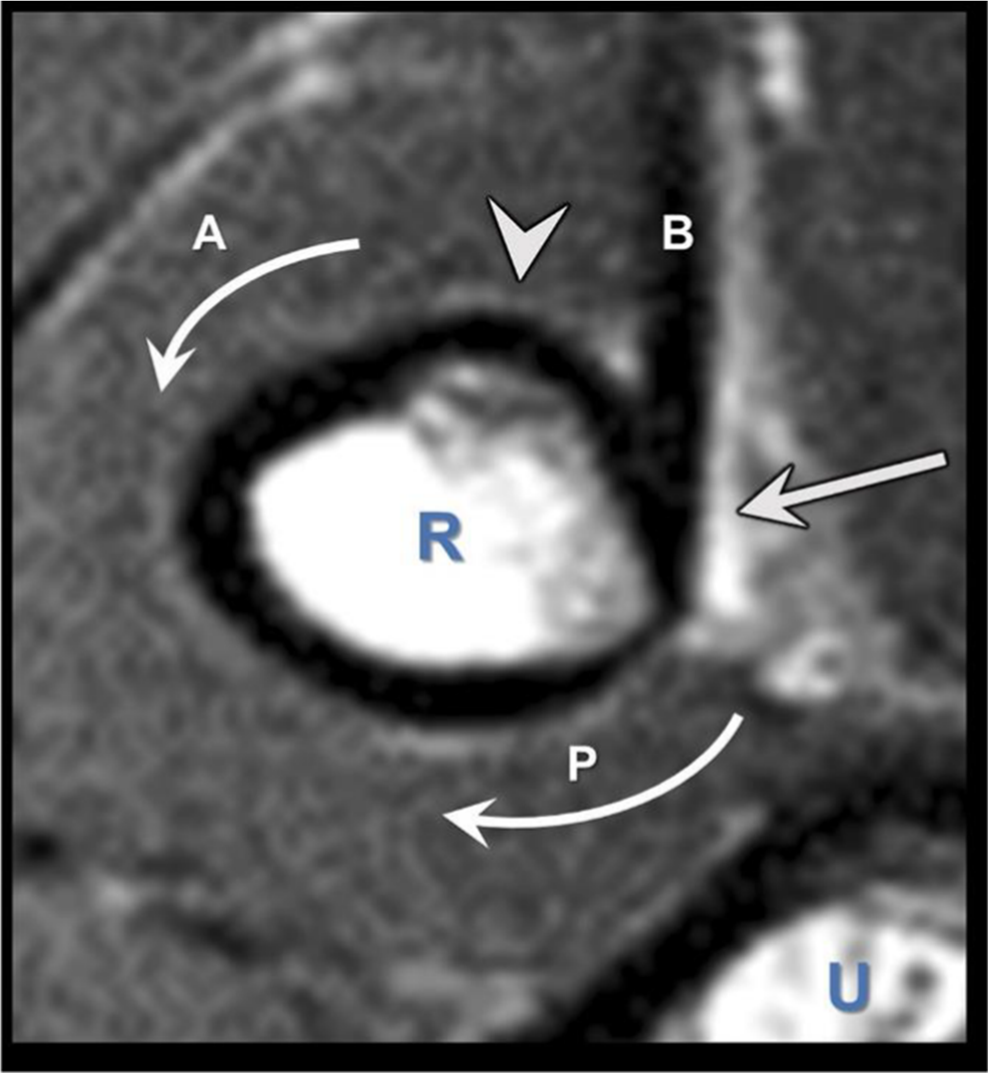
The magnetic resonance imaging (MRI) axial view through the insertion of the distal biceps. The arrow shows the biceps tendon (B). The anterior (A) and posterior (P) curved arrows show the arc of the radial tuberosity. The protuberance (arrowhead) of the radial tuberosity is found anterior to the tendon insertion and is thought to function as a mechanical cam in increasing the supination moment of the biceps. R, radius; U, ulna. (Reprinted from *J Bone and Joint Surgery*, 2015, doi.org/10.2106/JBJS.N.01221, Schmidt CC, Brown BT, Williams BG, Rubright JH, Schmidt DL, Pic AC, Nakashian MR, Schimoler PJ, Miller MC with permission from Wolters Kluwer (The Importance of Preserving the Radial Tuberosity During Di... : JBJS (lww.com))

In the majority of patients, the biceps brachii muscle originates and inserts as two separate heads (Fig. 1). In 13% of patients, there is bifid head with two separate and distinct heads, while in the other 87%, the heads are joined, but are easily separated [30, 31]. The long head runs lateral to the short head up to the myotendinous junction; then, the tendon externally rotates 90° as it traverses the bicipital tunnel, positioning the long head proximal to the short head on the insertion footprint [1, 21]. The distal biceps tendon inserts to the posterior aspect of the radial tuberosity [1, 21, 25, 32] (Fig. 1). The center of the long head insertion is slightly more posterior than the short head insertion, giving it a better lever arm to be a stronger supinator [25]. Otherwise, the short head insertion is more distal than the long head, generating 15% more flexion load than the long head. [25]. The specialized protuberance of the radial tuberosity lies just anterior to the biceps insertion and acts as a supination cam (Fig. 2) [2, 22, 24, 27].

## Biomechanics

**Figure 3:**
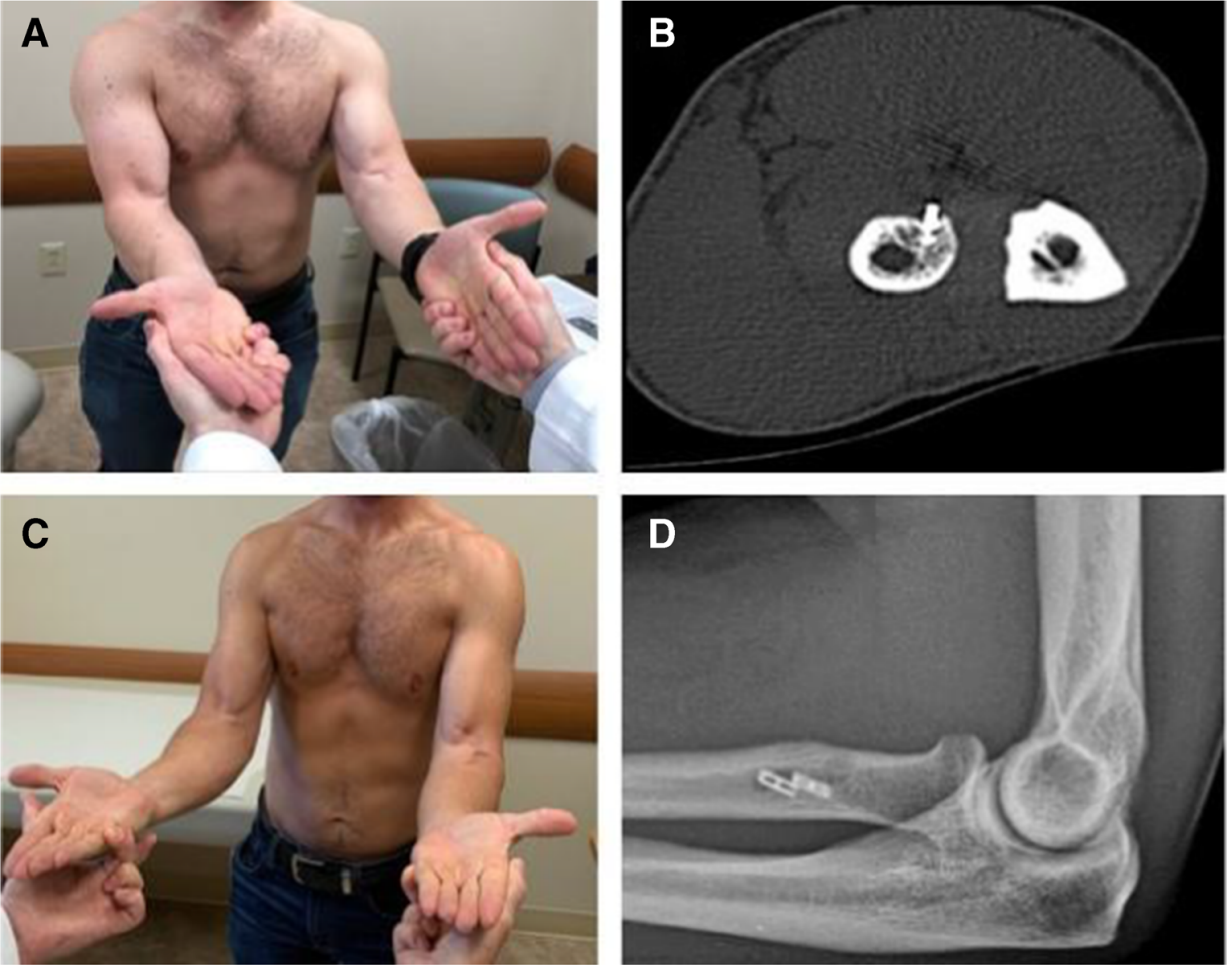
**A** Photograph of the patient that shows weakness in terminal supination, after distal biceps repair through an anterior approach. This prevents him from competing as a marksman as he cannot stabilize his rifle. **B** Axial CT scan showing distal biceps anchors placed anterior to the proutuberance, decreasing the moment arm of the tendon, and causing his weakness. **C** Post-operative photograph showing the improvement in the supination strength after revision repair in an anatomic position though a posterior approach. **D** Post-operative x-ray showing the anatomic repair of both distal biceps tendon heads with 2 intramedullary unicortical buttons

Two biomechanical studies have shown that an anterior reattachment site decreases supination torque by 15% in neutral and by 40% in 45° of supination (*p*=.01) and reduces supination moment arm by 27% in neutral and by 97% in 60° of supination (*p*<.05) [26, 29]. The protuberance at the radial tuberosity also plays a role in forearm supination as a cam (Fig. 2). Schmidt et al. [27] noted in their biomechanical study burring a socket/trough in the tuberosity results in a 27% loss (*p*=.04) in the biceps supination moment arm in a supinated forearm position. These mechanical studies imply that restoration of the insertional anatomy plays a significant role in restoration of pre-injury flexion and supination strength of the forearm. Only two clinical studies have looked at repair site location. Schmidt et al. [28] reported 19 patients who underwent distal biceps repair using an anterior approach. Post-operative MRI showed an insertion site angle of the repaired tendons was 73° more anterior than the uninjured controls (*p*<.001), and at 60° of forearm supination, supination strength was 67% of the uninjured side (*p*<.01) [28]. Another series of 27 patients underwent distal bicep repair via anterior approach; post-operative CT showed that the average suture anchor placement was 50° radial to the apex of the tuberosity [33]. Testing showed flexion strength of the repaired side was equal (97–106%) to that of the normal side, but supination strength (80–86%) and work (66–75%) performed were both weaker on the repaired side (66–75%; *p*<.05) [33]. In the contralateral side of the dominant arm of a non-athlete person, supination strength deficits may pass undetected. This may not be the case for athletes or laborers who require high level skills with the upper extremity. A clinical case is shown in Figure 3 emphasizing the importance of anatomic repair. This patient was a professional marksman who had difficulty stabilizing the gunstock of his rifle after anterior repair. After repair revision, patient was able to participate in his marksmanship.

## Nonoperative Management

Nonoperative management of DBTR is reserved for sedentary, low-demand patients or patients not medically fit for surgery. If treated nonsurgically, patients can expect painless function with diminished strength, especially in supination [14]. In a retrospective study, 20 cases of DBTR treated nonoperatively were studied [34]. Compared to a historical control group, there was a significant difference in mean supination strength (74% versus 101%; *p*=0.002), but mean flexion strength (88% compared with 97%; *p=*0.164) was not significantly different. In the same series, the average reported Disabilities of the Arm, Shoulder, and Hand (DASH) score was 14 (US population avg. is 10.1), but 38% (6/16) of the patients reported weakness turning a screwdriver and 50% (8/16) reported difficulty lifting heavy objects, noting that DASH score does not measure the functional loss following a DBTR [34, 35]. Another clinical study evaluated 9 patients with nonrepaired DBTR and they found a 30% loss in flexion peak torque and a 50% loss in supination peak torque [36]. In another study, 23 male patients with complete DBTR were subject to isometric supination strength testing [10]. They found that patients with complete injuries to the distal biceps can lose up to 60% of supination strength. To avoid these functional deficits, surgical management is warranted.

For athletes, the downside of surgery is missing time. In studies performed on NFL players undergoing distal biceps repair, all patients lost the rest of the season [37••, 38••]. Some athletes can possibly be candidates for limited nonoperative management if they would like to finish the season. A case-control study investigated delayed repair of distal biceps tendon [39•]. Sixteen patients underwent delayed repair of distal biceps (>21 days) and were watched with an acute repair control group. Complications occurred in 63% of patients in the delayed cohort versus 29% in the acute cohort (*p* = .04); however, 90% of the delayed cohort’s complications consisted of transient paresthesia and PROs showed no difference between the groups (*p*>.05) [39•]. The case can be made for certain athletes to complete the season and have a delayed repair.

## Operative Management

Most complete DBTR are treated surgically, especially in active patients and athletes. Surgical repair aims to restore the distal tendon to its anatomic footprint. Surgical approaches include anterior approach (single incision) and posterior approach (double incision), and both have advantages and disadvantages. Due to a more extensive anterior dissection, the anterior approach has a higher incidence of lateral antebrachial cutaneous nerve (LACN) palsies [4, 5]. However, these injuries tend to be self-resolving with non-permanent dysfunction. Boyd and Anderson [40] developed the posterior approach to avoid these neurologic injuries. The posterior approach had been associated with radio-ulna synostosis and posterior interosseous nerve (PIN) palsy. Since first described, multiple modifications have been made to avoid these complications [41]. Recent literature shows a low incidence of symptomatic heterotopic ossification (HO), due to avoiding the ulna and prescribing indomethacin 75mg for 14 days. A prospective cohort study comparing both approaches observed 1 patient (1/9) in the anterior group with motion-limiting HO compared with none (0/10) in the posterior group [42]. The patients were given indomethacin 25 mg 3 times a day and misoprostol 200 mcg twice a day for 2 weeks.

A systematic review of 22 studies included 494 patients (498 elbows) who underwent distal biceps tendon repair [20]. Their series reported an overall complication rate of 24.5%, with no differences reported between anterior approach (23.9%) and posterior approach (25.7%; *p*=0.32). Overall, LACN neurapraxia was the most common complication and it was more common in the patients who underwent an anterior approach (11.6%) versus posterior approach (5.8%; *p*=0.02). HO occurred in 4.4% of the patients and was more common in the patients who underwent a posterior approach (7%) compared with the patients who underwent an anterior approach (3.1%; *p*=0.06); however, medical prophylaxis was not given.

Multiple cadaveric studies have investigated the restoration of the anatomical footprint using cadaveric models. Hasan et al. [23] showed that the posterior approach was more reliable than the anterior approach (73.4% vs 9.7%) at creating a virtual tunnel at the anatomic footprint. Another cadaveric study showed that the posterior approach with double suture anchor restored the footprint in a more posterior and anatomic position than the anterior approach (*p*=.001) [43]. Also, Forthman et al. [22] reported that 35% of cadaveric specimens investigated had a more pronated tuberosity, prohibiting anatomic repair of the tendon with current anterior approach techniques. All of these studies support that the posterior approach is more reliable at restoring the anatomical footprint of the distal biceps.

In a randomized controlled trial, Grewal et al. [4] reported no difference in DASH score, American Shoulder and Elbow (ASES) elbow score, and Patient-Rated Elbow Evaluation (PREE) when comparing patients treated with anterior approach to posterior approach at 2-year follow-up. Although there were no differences in isometric extension, pronation, or supination strength, they used a drill hole technique with a through on the tuberosity in the posterior approach group. Also, they did not evaluate repair site position in this study. A prospective study compared 15 patients who underwent distal biceps repair through a posterior approach to 17 randomized patients who underwent anterior approach. They found that patients in the posterior group had better supination strength at 60° of supination (*p*=0.027) [44]. Also, anatomic reinsertion of the tendon (*β*=1.159; *p*<0.001), posterior approach (*β*=0.484; *p*=0.043), and limited supinator muscle fat (*β*=0.360; *p* =0.013) were significant predictors of restoration of supination strength in 60° [45].

## Our Preferred Technique

**Figure 4:**
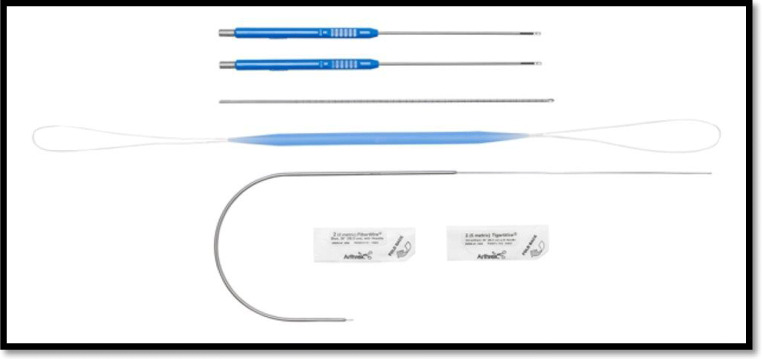
Arthrex distal biceps repair kit. Includes 2 intramedullary cortical buttons, a 3.2-mm drill bit, a dilator, a curved biceps passer, and 2 double-loaded fiber wires of different colors

**Figure 5:**
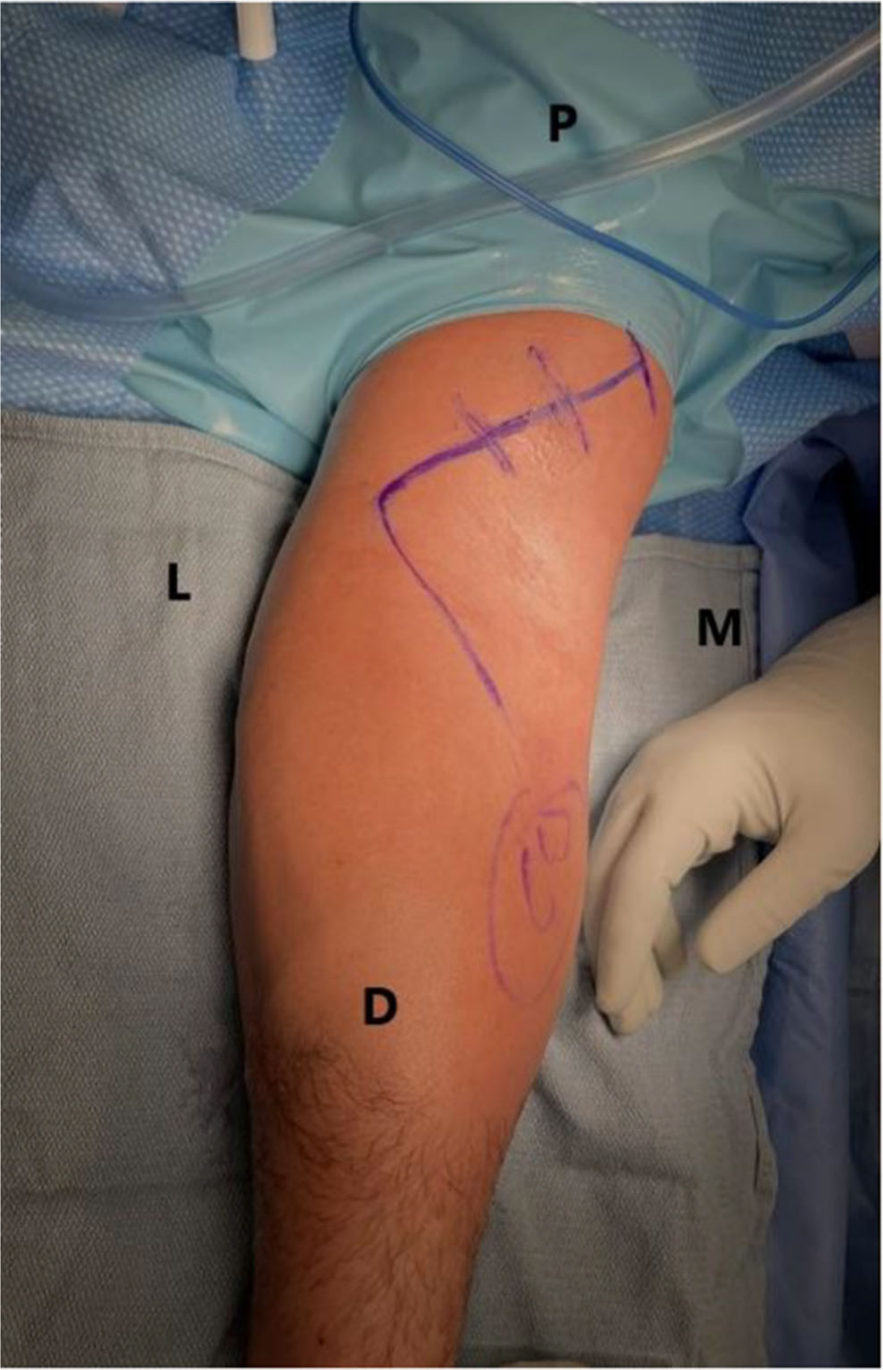
An anterior oblique incision is used to identify the tendon. The incision can be extended proximally for retracted tendons and distally when the lacertus fibrosus is intact and the tendon lies more distally

**Figure 6:**
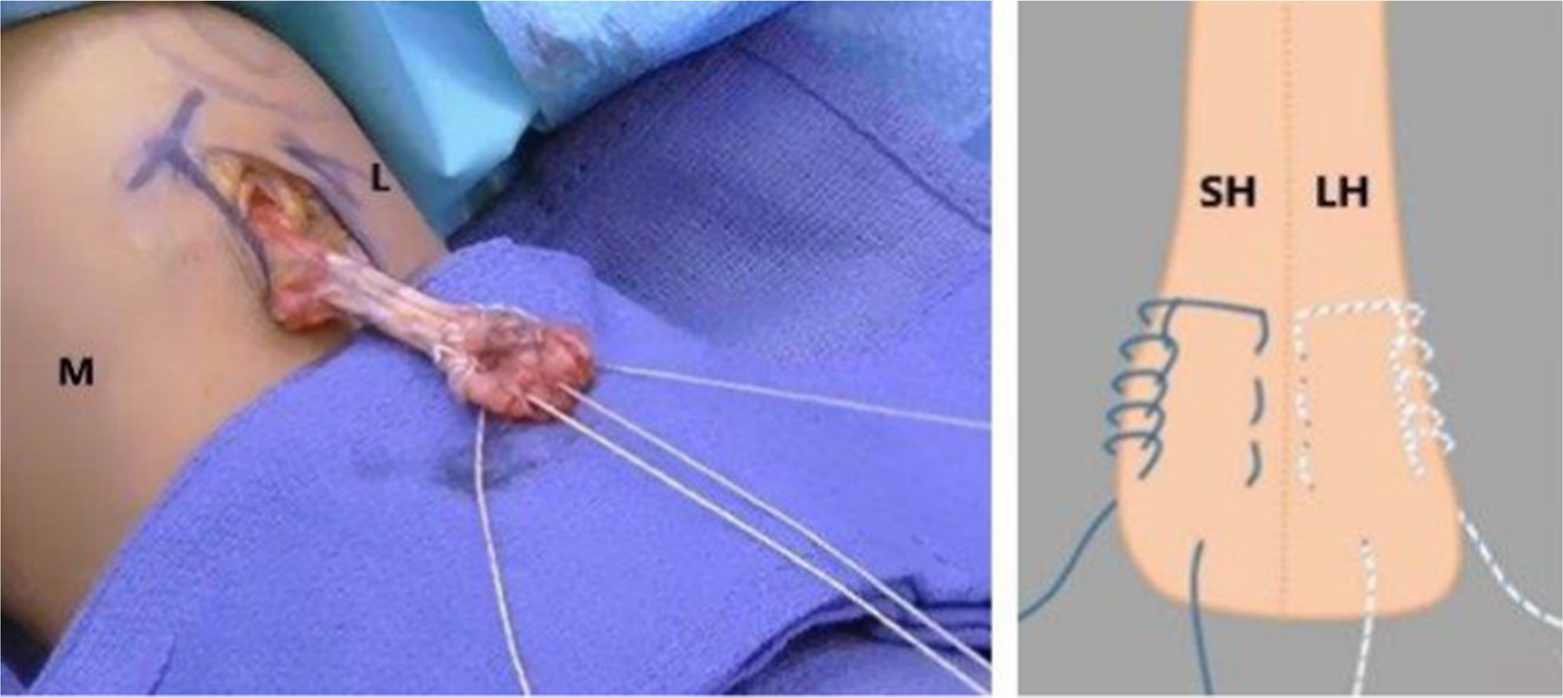
On the left is an intraoperative photograph of the four-throw modified Krackow stitch being placed in each head of the distal biceps tendon. On the right is an illustration of the Krackow stitch. (Reprinted with modifications from *Advanced Reconstruction Elbow* 2, 2016, ISBN: 978-1-62-552546-8, Ring D, Steinmann ST with permission from Wolters Kulwer)

The Arthrex Distal Biceps Repair Kit (Arthrex, Naples, FL, USA) has been recently introduced and it provides all the tools for an anatomic posterior approach repair using 2 cortical intramedullary buttons (Fig. 4). An oblique anterior incision is used to find the tendon proximally. If the lacertus fibrosus is intact, the incision can be extended distally around the elbow flexion crease laterally (Fig. 5). Identify the LACN and neurolysis may be needed if the nerve is surrounded by scar tissue. The distal tendon is often encased in pseudo tendon. The scar is removed from the distal tendon end to expose the short and long heads to ensure proper tendon alignment. A four-throw modified Krackow whipstitch is placed in each head. The outside limb of the stitch is locked, and the stitch ends 5 to 10 mm from the tendon end on the dorsal side. The central limb of the suture on each head is placed in a simple running pattern and finishes in the center of each head on the palmar side (Fig. 6A, B).

**Figure 7:**
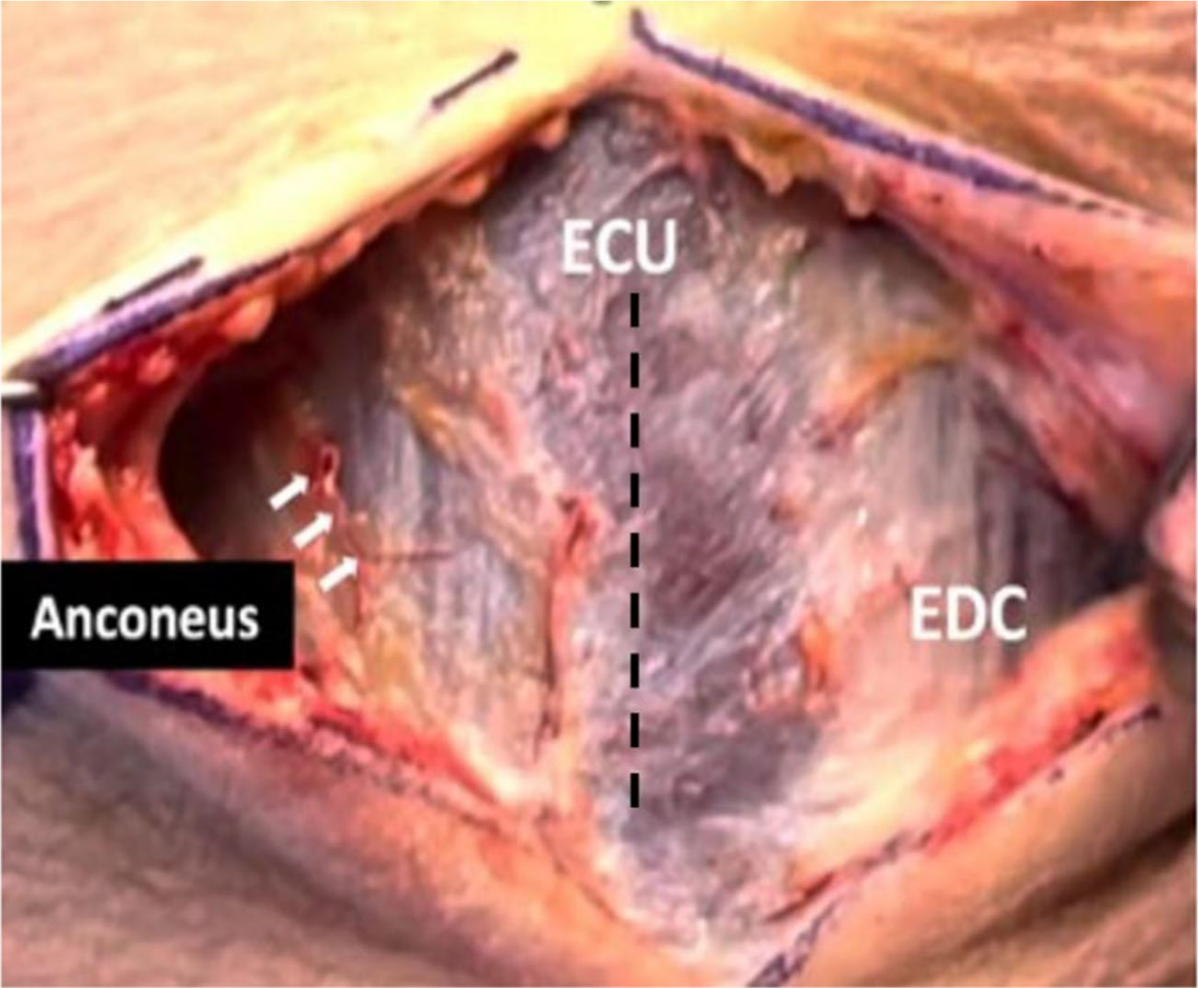
Intraoperative photograph. The ECU will be splitted to access the supinator muscle (dashed line). ECU, extensor carpi ulnaris; EDC, extensor digitorum communis

**Figure 8:**
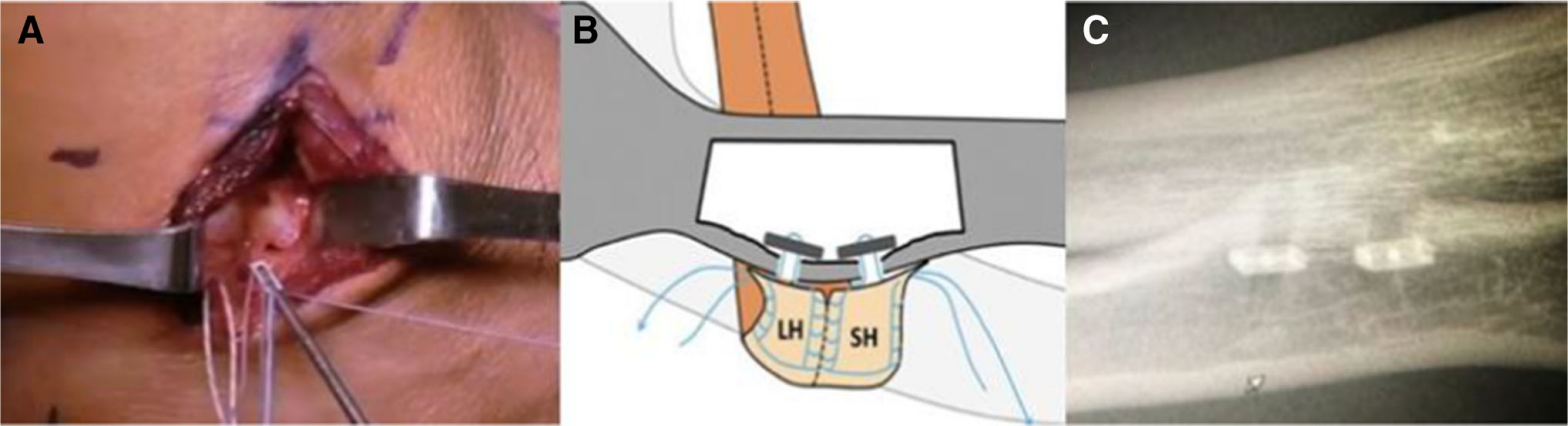
**A** Intraoperative photograph of the cortical button and the drill holes at the anatomic footprint of both heads. **B** Diagram of the cortical buttons securing to the heads of the distal biceps in an onlay fashion. **C** Post-operative x-rays show the anatomic repair of both heads of the distal biceps with 2 intermedullary cortical buttons. (Reprinted from *J Hand Surgery*, 2013, doi.org/10.1016/j.jhsa.2013.01.042, Schmidt CC, Jarrett CD, Brown BT with permission from Elsevier) (The Distal Biceps Tendon - ScienceDirect)

The radial tuberosity is exposed posteriorly through a 3- to 4-cm incision starting 6 cm from the olecranon and 1.5 cm dorsal to the ulnar ridge. With the patient’s forearm in pronation, the extensor carpi ulnaris (ECU) muscle is split in line with its fibers (Fig. 7). The supinator muscle is incised directly over the tuberosity. The bicipitoradial bursa and tendon remnants are cleared from the tuberosity with a rongeur. A curved biceps passer is passed from back to front; then, a dilator is used to create the path of the bicipital tunnel. The tendon is passed from volar to dorsal along its native path. Forearm supination aids in tendon passage. The tendon is externally 90° so that the short head is repaired distal to the long head. With the use of a 3.2-mm biceps drill pin, cortical drill holes are made at the center of the footprint of each head about 1 cm apart (Fig. 8A). The docking limb of each suture is loaded to a biceps cortical button. The buttons for both the short head and the long head are placed in their respective drill holes (Fig. 8B). To ensure, the buttons are flipped and the tendon is cycled. The docking stitches are pulled to compress their respective biceps heads against the footprint and the sutures are tied. Two cortical intramedullary buttons provide the same load to failure as the native tendon [45] and avoid posterior interosseous nerve injuries. Button position is confirmed by fluoroscopy (Fig. 8C). The skin is closed in layers [46].

After surgery, the elbow is immobilized in a posterior splint in neutral position for 2 weeks. At 2 weeks, sutures are removed, and the patient is transitioned to a custom-made splint. Passive and active motions of the forearm are encouraged but lifting more than 5 pounds is not allowed. At 6 weeks, all restrictions are lifted, and patients can start strengthening exercises. We prefer to protect the patient in the initial 6 weeks because all acute repair failures reported in the literature occur within the first 6 weeks after surgery [35]. To avoid heterotopic ossification (HO), all patients take 75mg of indomethacin daily for 2 weeks. Return to play for contact sports is allowed at 12 weeks without bracing.

## Return to Play After Distal Biceps Tendon Rupture

There was a paucity of literature regarding return to play and outcomes of athletes following a DBTR. Previous articles have evaluated performance and return to work in certain groups, but not in athletes. In a systematic review, Rubinger et al. [18] evaluated 40 articles that included a total of 1270 patients with 1280 DBTR. The mean age of patients was 45.38 years, and 97% were male. The mean follow-up time was 30 months (range, 6–84 months). After surgical distal biceps repair, 1128 (89%) of the patients were able to fully return to work without any modification of duties. The mean time to return to work was 14.27±0.52 weeks. One prior study evaluating a military population reported excellent clinical outcomes with 15.2% (*n*=44) overall complications, 7.5% (*n*=16) LACN neuropraxia, and 2.7% (*n*=7) re-tears and 96.6% return to pre-injury military duties without restrictions [18]. Also, a study investigating a military population observed excellent clinical outcomes and 96.6% return to pre-injury military duties without restrictions [19].

Regarding athletes undergoing distal biceps tendon repair, one study reported 10 athletes with an average of 40 years old that underwent surgical repair of the distal biceps, and all returned to unrestricted activities without pain [47]. Eight of those patients were categorized as weightlifters but their level of competition was poorly defined. They also reported isometric strength testing, showing no difference in supination or flexion strength and decreased flexion endurance [47]. A recent study evaluated return to sport and weightlifting after distal biceps tendon repair [48•]. They retrospectively evaluated 61 patients who endorsed activity and underwent distal biceps repair. Mean average age at surgery was 45.7±8.8 years and the average follow-up was 38.7±6.7 years. Fifty-seven patients (93.4%) returned to sports at any level and 40 (65.6%) returned to same or higher intensity level. Mean time to return to sport was 6.0±2.8 months. They also assessed for single repetition maximum (1RM) biceps curl and 10RM biceps curls and found no difference between pre-operative and post-operative values (*p*=.757 and .950 respectively).

In NFL players, this injury is more likely to occur in offensive and defensive linemen, most likely due to the use of the upper extremity in high energy [37••, 38••]. Pagani et al. [37••] examined data for National Football League (NFL) players who underwent surgical repair of DBTR during a 20-year period. Their study included 25 cases (22 patients) and matched controls based on player position, experience, and performance statistics were evaluated. Twenty-one cases (84%) were able to return to play at least one game, with an average number of days to return to sports of 321±45 days. Compared to their matched control cohort, players that underwent surgical repair had significantly shorter careers after surgery (3.4±2.0 years vs 2.8±2.0 years, *p*=0.049) and played significantly less games per season (13±2.3 years vs 11±4.0 years, *p*=0.02) [37••]. However, the authors suggest that other factors may have affected this. Most players were late in their careers at the time of injury and were part of the NFL average career length. There was no significant difference in performance scores post-surgery compared to their matched controls [36]. Another recent study retrospectively analyzed NFL players [38••]. Thirty-five NFL players were identified for the study and 94% (33/35) were able to return to sport at an average of 11.5 (±4.1) months. Offensive lineman undergoing surgery played less games per season compared to the control group (*p* = 0.04), but mean career length, or games per season, did not differ for post-surgical versus control group (*p* >0.05) for all other positions. The average seasons post-surgery compared to a matched control group was not found to not be significant (*p* > 0.05). Performance scores within skill players were not statistically significant between post-operative and matched control groups (*p*> 0.05) [38••].

## Conclusions

Distal biceps tendon ruptures (DBTR) are a debilitating injury that requires surgical fixation in active patients, especially in athletes. Anatomic repair through a posterior approach has been shown to improve supination endurance biomechanically, although controversy remains in its clinical significance. High-performing athletes require high demands of their body and the senior author advocates for a posterior approach to maximize their function post injury. A non-anatomic repair would result in diminished supination endurance, especially when supinating away from the body. The literature on elite athletes shows there is a high likelihood that NFL players will return to play (84–94%) and will perform at the same level as their matched peers and their career length may not be decreased.

## Supplementary Information

ESM 1 (PDF 103 kb)

ESM 2 (PDF 154 kb)

ESM 3 (PDF 158 kb)

ESM 4 (PDF 50 kb)
