# Supplementary material for: Return to Play After Distal Biceps Tendon Repair
Source: Curr Rev Musculoskelet Med. 2022 Feb 23;15(2):65–74. doi: 10.1007/s12178-022-09742-x (PMC9076792; doi:10.1007/s12178-022-09742-x)
Supplement: Supplementary file 4 — (PDF 50 kb) [file 12178_2022_9742_MOESM4_ESM.pdf]

CCC Marketplace™

This is a License Agreement between Christopher Schmidt ("User") and Copyright Clearance Center, Inc. ("CCC") on behalf of the Rightsholder identified in the order details below. The license consists of the order details, the CCC Terms and Conditions below, and any Rightsholder Terms and Conditions which are included below.

All payments must be made in full to CCC in accordance with the CCC Terms and Conditions below.

**Order Date**	28-Jul-2021	**Type of Use**	Republish in a journal/magazine
**Order License ID**	1136529-1	**Publisher**	W.B./SAUNDERS CO.
**ISSN**	0363-5023	**Portion**	Image/photo/illustration

LICENSED CONTENT

**Publication Title**	The Journal of hand surgery	**Rightsholder**	Elsevier Science & Technology Journals
**Article Title**	The distal biceps tendon.	**Publication Type**	Journal
**Author/Editor**	AMERICAN SOCIETY FOR SURGERY OF THE HAND.	**Start Page**	811
		**End Page**	821
**Date**	01/01/1976	**Issue**	4
**Language**	English	**Volume**	38
**Country**	United States of America		

REQUEST DETAILS

**Portion Type**	Image/photo/illustration	**Distribution**	Worldwide
**Number of images / photos / illustrations**	1	**Translation**	Original language of publication
**Format (select all that apply)**	Print, Electronic	**Copies for the disabled?**	No
		**Minor editing privileges?**	No
**Who will republish the content?**	Author of requested content	**Incidental promotional use?**	No
**Duration of Use**	Life of current edition	**Currency**	USD
**Lifetime Unit Quantity**	Up to 999		
**Rights Requested**	Main product		

NEW WORK DETAILS

**Title**	Return to play after distal biceps tendon repair	**Publisher imprint**	N/A
**Author**	Luis Carrazana-Suarez	**Expected publication date**	2021-10-01
**Publication**	Current Reviews in Musculoskeletal Medicine	**Expected size (number of pages)**	10
**Publisher**	Springer	**Standard identifier**	N/A

ADDITIONAL DETAILS

**Order reference number**	N/A

**The requesting person / organization to appear on the license**	Christopher Schmidt

REUSE CONTENT DETAILS

**Title, description or numeric reference of the portion(s)**	Figure 10 C	**Title of the article/chapter the portion is from**	The distal biceps tendon.
**Editor of portion(s)**	Schmidt, Christopher C., MD; Jarrett, Claudius D., MD; Brown, Brandon T., BS	**Author of portion(s)**	Schmidt, Christopher C., MD; Jarrett, Claudius D., MD; Brown, Brandon T., BS
**Volume of serial or monograph**	38	**Issue, if republishing an article from a serial**	4
**Page or page range of portion**	811-821	**Publication date of portion**	2013-04-01

RIGHTSHOLDER TERMS AND CONDITIONS

Elsevier publishes Open Access articles in both its Open Access journals and via its Open Access articles option in subscription journals, for which an author selects a user license permitting certain types of reuse without permission. Before proceeding please check if the article is Open Access on http://www.sciencedirect.com and refer to the user license for the individual article. Any reuse not included in the user license terms will require permission. You must always fully and appropriately credit the author and source. If any part of the material to be used (for example, figures) has appeared in the Elsevier publication for which you are seeking permission, with credit or acknowledgement to another source it is the responsibility of the user to ensure their reuse complies with the terms and conditions determined by the rights holder. Please contact permissions@elsevier.com with any queries.

CCC Terms and Conditions

1. Description of Service; Defined Terms. This Republication License enables the User to obtain licenses for republication of one or more copyrighted works as described in detail on the relevant Order Confirmation (the "Work(s)"). Copyright Clearance Center, Inc. ("CCC") grants licenses through the Service on behalf of the rightsholder identified on the Order Confirmation (the "Rightsholder"). "Republication", as used herein, generally means the inclusion of a Work, in whole or in part, in a new work or works, also as described on the Order Confirmation. "User", as used herein, means the person or entity making such republication.

2. The terms set forth in the relevant Order Confirmation, and any terms set by the Rightsholder with respect to a particular Work, govern the terms of use of Works in connection with the Service. By using the Service, the person transacting for a republication license on behalf of the User represents and warrants that he/she/it (a) has been duly authorized by the User to accept, and hereby does accept, all such terms and conditions on behalf of User, and (b) shall inform User of all such terms and conditions. In the event such person is a "freelancer" or other third party independent of User and CCC, such party shall be deemed jointly a "User" for purposes of these terms and conditions. In any event, User shall be deemed to have accepted and agreed to all such terms and conditions if User republishes the Work in any fashion.

3. Scope of License; Limitations and Obligations.

    3.1. All Works and all rights therein, including copyright rights, remain the sole and exclusive property of the Rightsholder. The license created by the exchange of an Order Confirmation (and/or any invoice) and payment by User of the full amount set forth on that document includes only those rights expressly set forth in the Order Confirmation and in these terms and conditions, and conveys no other rights in the Work(s) to User. All rights not expressly granted are hereby reserved.

    3.2.

General Payment Terms: You may pay by credit card or through an account with us payable at the end of the month. If you and we agree that you may establish a standing account with CCC, then the following terms apply: Remit Payment to: Copyright Clearance Center, 29118 Network Place, Chicago, IL 60673-1291. Payments Due: Invoices are payable upon their delivery to you (or upon our notice to you that they are available to you for downloading). After 30 days, outstanding amounts will be subject to a service charge of 1-1/2% per month or, if less, the maximum rate allowed by applicable law. Unless otherwise specifically set forth in the Order Confirmation or in a separate written agreement signed by CCC, invoices are due and payable on "net 30" terms. While User may exercise the rights licensed immediately upon issuance of the Order Confirmation, the license is automatically revoked and is null and void, as if it had never been issued, if complete payment for the license is not received on a timely basis either from User directly or through a payment agent, such as a credit card company.

3.3. Unless otherwise provided in the Order Confirmation, any grant of rights to User (i) is "one-time" (including the editions and product family specified in the license), (ii) is non-exclusive and non-transferable and (iii) is subject to any and all limitations and restrictions (such as, but not limited to, limitations on duration of use or circulation) included in the Order Confirmation or invoice and/or in these terms and conditions. Upon completion of the licensed use, User shall either secure a new permission for further use of the Work(s) or immediately cease any new use of the Work(s) and shall render inaccessible (such as by deleting or by removing or severing links or other locators) any further copies of the Work (except for copies printed on paper in accordance with this license and still in User's stock at the end of such period).

3.4. In the event that the material for which a republication license is sought includes third party materials (such as photographs, illustrations, graphs, inserts and similar materials) which are identified in such material as having been used by permission, User is responsible for identifying, and seeking separate licenses (under this Service or otherwise) for, any of such third party materials; without a separate license, such third party materials may not be used.

3.5. Use of proper copyright notice for a Work is required as a condition of any license granted under the Service. Unless otherwise provided in the Order Confirmation, a proper copyright notice will read substantially as follows: "Republished with permission of [Rightsholder's name], from [Work's title, author, volume, edition number and year of copyright]; permission conveyed through Copyright Clearance Center, Inc. " Such notice must be provided in a reasonably legible font size and must be placed either immediately adjacent to the Work as used (for example, as part of a by-line or footnote but not as a separate electronic link) or in the place where substantially all other credits or notices for the new work containing the republished Work are located. Failure to include the required notice results in loss to the Rightsholder and CCC, and the User shall be liable to pay liquidated damages for each such failure equal to twice the use fee specified in the Order Confirmation, in addition to the use fee itself and any other fees and charges specified.

3.6. User may only make alterations to the Work if and as expressly set forth in the Order Confirmation. No Work may be used in any way that is defamatory, violates the rights of third parties (including such third parties' rights of copyright, privacy, publicity, or other tangible or intangible property), or is otherwise illegal, sexually explicit or obscene. In addition, User may not conjoin a Work with any other material that may result in damage to the reputation of the Rightsholder. User agrees to inform CCC if it becomes aware of any infringement of any rights in a Work and to cooperate with any reasonable request of CCC or the Rightsholder in connection therewith.

4. Indemnity. User hereby indemnifies and agrees to defend the Rightsholder and CCC, and their respective employees and directors, against all claims, liability, damages, costs and expenses, including legal fees and expenses, arising out of any use of a Work beyond the scope of the rights granted herein, or any use of a Work which has been altered in any unauthorized way by User, including claims of defamation or infringement of rights of copyright, publicity, privacy or other tangible or intangible property.

5. Limitation of Liability. UNDER NO CIRCUMSTANCES WILL CCC OR THE RIGHTSHOLDER BE LIABLE FOR ANY DIRECT, INDIRECT, CONSEQUENTIAL OR INCIDENTAL DAMAGES (INCLUDING WITHOUT LIMITATION DAMAGES FOR LOSS OF BUSINESS PROFITS OR INFORMATION, OR FOR BUSINESS INTERRUPTION) ARISING OUT OF THE USE OR INABILITY

TO USE A WORK, EVEN IF ONE OF THEM HAS BEEN ADVISED OF THE POSSIBILITY OF SUCH DAMAGES. In any event, the total liability of the Rightsholder and CCC (including their respective employees and directors) shall not exceed the total amount actually paid by User for this license. User assumes full liability for the actions and omissions of its principals, employees, agents, affiliates, successors and assigns.

6. Limited Warranties. THE WORK(S) AND RIGHT(S) ARE PROVIDED "AS IS". CCC HAS THE RIGHT TO GRANT TO USER THE RIGHTS GRANTED IN THE ORDER CONFIRMATION DOCUMENT. CCC AND THE RIGHTSHOLDER DISCLAIM ALL OTHER WARRANTIES RELATING TO THE WORK(S) AND RIGHT(S), EITHER EXPRESS OR IMPLIED, INCLUDING WITHOUT LIMITATION IMPLIED WARRANTIES OF MERCHANTABILITY OR FITNESS FOR A PARTICULAR PURPOSE. ADDITIONAL RIGHTS MAY BE REQUIRED TO USE ILLUSTRATIONS, GRAPHS, PHOTOGRAPHS, ABSTRACTS, INSERTS OR OTHER PORTIONS OF THE WORK (AS OPPOSED TO THE ENTIRE WORK) IN A MANNER CONTEMPLATED BY USER; USER UNDERSTANDS AND AGREES THAT NEITHER CCC NOR THE RIGHTSHOLDER MAY HAVE SUCH ADDITIONAL RIGHTS TO GRANT.

7. Effect of Breach. Any failure by User to pay any amount when due, or any use by User of a Work beyond the scope of the license set forth in the Order Confirmation and/or these terms and conditions, shall be a material breach of the license created by the Order Confirmation and these terms and conditions. Any breach not cured within 30 days of written notice thereof shall result in immediate termination of such license without further notice. Any unauthorized (but licensable) use of a Work that is terminated immediately upon notice thereof may be liquidated by payment of the Rightsholder's ordinary license price therefor; any unauthorized (and unlicensable) use that is not terminated immediately for any reason (including, for example, because materials containing the Work cannot reasonably be recalled) will be subject to all remedies available at law or in equity, but in no event to a payment of less than three times the Rightsholder's ordinary license price for the most closely analogous licensable use plus Rightsholder's and/or CCC's costs and expenses incurred in collecting such payment.

8. Miscellaneous.

    8.1. User acknowledges that CCC may, from time to time, make changes or additions to the Service or to these terms and conditions, and CCC reserves the right to send notice to the User by electronic mail or otherwise for the purposes of notifying User of such changes or additions; provided that any such changes or additions shall not apply to permissions already secured and paid for.

    8.2. Use of User-related information collected through the Service is governed by CCC's privacy policy, available online here:https://marketplace.copyright.com/rs-ui-web/mp/privacy-policy

    8.3. The licensing transaction described in the Order Confirmation is personal to User. Therefore, User may not assign or transfer to any other person (whether a natural person or an organization of any kind) the license created by the Order Confirmation and these terms and conditions or any rights granted hereunder; provided, however, that User may assign such license in its entirety on written notice to CCC in the event of a transfer of all or substantially all of User's rights in the new material which includes the Work(s) licensed under this Service.

    8.4. No amendment or waiver of any terms is binding unless set forth in writing and signed by the parties. The Rightsholder and CCC hereby object to any terms contained in any writing prepared by the User or its principals, employees, agents or affiliates and purporting to govern or otherwise relate to the licensing transaction described in the Order Confirmation, which terms are in any way inconsistent with any terms set forth in the Order Confirmation and/or in these terms and conditions or CCC's standard operating procedures, whether such writing is prepared prior to, simultaneously with or subsequent to the Order Confirmation, and whether such writing appears on a copy of the Order Confirmation or in a separate instrument.

    8.5. The licensing transaction described in the Order Confirmation document shall be governed by and construed under the law of the State of New York, USA, without regard to the principles thereof of conflicts of law. Any case, controversy, suit, action, or proceeding arising out of, in connection with, or related to such licensing transaction shall be brought, at CCC's sole discretion, in any federal or state court located in the County of New York, State of New York, USA, or in any federal or state court whose geographical

jurisdiction covers the location of the Rightsholder set forth in the Order Confirmation. The parties expressly submit to the personal jurisdiction and venue of each such federal or state court.If you have any comments or questions about the Service or Copyright Clearance Center, please contact us at 978-750-8400 or send an e-mail to support@copyright.com.

v 1.1
